# Sex-specific dysregulation of cysteine oxidation and the methionine and folate cycles in female cystathionine gamma-lyase null mice: a serendipitous model of the methylfolate trap

**DOI:** 10.1242/bio.013433

**Published:** 2015-08-14

**Authors:** Hua Jiang, K. Joseph Hurt, Kelsey Breen, Sally P. Stabler, Robert H. Allen, David J. Orlicky, Kenneth N. Maclean

**Affiliations:** 1 Department of Pediatrics, University of Colorado School of Medicine, Aurora, CO 80045, USA; 2 Department of Obstetrics and Gynecology, University of Colorado School of Medicine, Aurora, CO 80045, USA; 3 Department of Medicine, University of Colorado School of Medicine, Aurora, CO 80045, USA; 4 Department of Pathology, University of Colorado School of Medicine, Aurora, CO 80045, USA

**Keywords:** Cystathionine γ-lyase, Hydrogen sulfide, Cystathionine β-synthase, Homocystinuria, Remethylation defect, Methionine synthase

## Abstract

In addition to its role in the endogenous synthesis of cysteine, cystathionine gamma-lyase (CGL) is a major physiological source of the vasorelaxant hydrogen sulfide. *Cgl* null mice are potentially useful for studying the influence of this compound upon vascular tone and endothelial function. Here, we confirm a previous report that female *Cgl* null mice exhibit an approximate 45-fold increase in plasma total homocysteine compared to wild type controls. This level of homocysteine is approximately 3.5-fold higher than that observed in male *Cgl* null mice and is essentially equivalent to that observed in mouse models of cystathionine beta synthase deficient homocystinuria. *Cgl* null mice of both sexes exhibited decreased expression of methylenetetrahydrofolate reductase and cysteinesulfinate decarboxylase compared to WT controls. Female *Cgl* null mice exhibited a sex-specific induction of betaine homocysteine S-methyltransferase and methionine adenosyltransferase 1, alpha and a 70% decrease in methionine synthase expression accompanied by significantly decreased plasma methionine. Decreased plasma cysteine levels in female *Cgl* null mice were associated with sex-specific dysregulation of cysteine dioxygenase expression. Comparative histological assessment between cystathionine beta-synthase and *Cgl* null mice indicated that the therapeutic potential of cystathionine against liver injury merits possible further investigation. Collectively, our data demonstrates the importance of considering sex when investigating mouse models of inborn errors of metabolism and indicate that while female *Cgl* null mice are of questionable utility for studying the physiological role of hydrogen sulfide, they could serve as a useful model for studying the consequences of methionine synthase deficiency and the methylfolate trap.

## INTRODUCTION

**Fig. 1.**
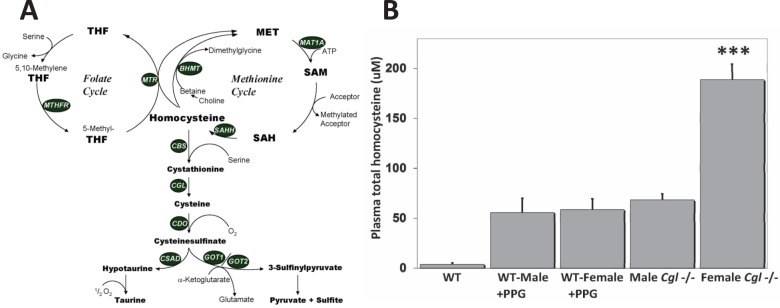
**Methionine, folate and cysteine metabolism in mammals.** (A) The transsulfuration and cysteine oxidation pathways and, methionine and folate cycles are shown. Betaine-homocysteine S-methyltransferase (BHMT), cystathionine β-synthase (CBS), cystathionine γ-lyase (CGL), cysteine dioxygenase (CDO), cysteinesulfinate decarboxylase (CSAD), glutamic-oxaloacetic transaminase (GOT), glycine N-methyltransferase (GNMT), methionine adenosyl transferase (MAT1A), methionine synthase (MTR), methylenetetrahydrofolate reductase (MTHFR), S-adenosyl homocysteine hydrolase (SAHH). (B) *Cgl* null female but not male mice exhibit severely elevated levels of plasma tHcy. Plasma levels of tHcy in wild type (WT) mice plus and minus treatment with the CGL specific inhibitor PPG and untreated male and female *Cgl* null mice. Values shown represent the mean±s.d., *n*=6, ***P<0.001.

Cystathionine γ-lyase (CGL) (EC4.4.1.1) is a pyridoxal-5′-phosphate-dependent enzyme that catalyzes the γ-cleavage of cystathionine [2-amino-4-(2-amino-2-carboxy-ethyl) thio-butanoic acid] to yield cysteine, α-ketobutyrate and ammonia as the terminal reaction of the transsulfuration pathway (Fig. 1A). Deficiency of CGL causes cystathionuria which appears to be an essentially benign condition (Kraus et al., 2009). In addition to a role in endogenous cysteine synthesis, CGL catalyzes an α,β-disulfide elimination reaction that results in the production of pyruvate, ammonia and thiocysteine. This latter compound may then react with other thiol compounds to generate hydrogen sulfide (H_2_S). Recently, interest in the regulation of CGL has increased as data has accumulated implicating H_2_S as an endogenous gasotransmitter associated with multiple diseases including pancreatitis, diabetes, hypertension and septic and hemorrhagic shock (Szabó, 2007). The expression of CGL at various locations in the vasculature in isolation from CBS, has led to speculation that synthesis of H_2_S by this enzyme is involved in the regulation of vascular tone. Previous work demonstrating that NaHS produces significant dose-dependent decreases in the spontaneous contractility of uterine strips from pregnant rats has indicated that CGL may be specifically involved in the regulation of vascular tone and contractility during pregnancy and childbirth (Sidhu et al., 2001). A previously described *Cgl* null mouse model (Yang et al., 2008) would seem an ideal system to investigate this hypothesis but a previous report has indicated that female Cgl null mice incur severely elevated plasma total homocysteine (tHcy). This compound has the potential to form mixed disulfides with the H_2_S donor compound cysteine and thus interfere with studies designed to assess the potential vasorelaxant role of endogenously generated H_2_S (Yang et al., 2008). The reason for this female-specific biochemical anomaly and possible changes to other relevant metabolites has to date, remained uninvestigated.

In the present study, we confirm that female *Cgl* null mice exhibit severely elevated tHcy which is some three-fold greater than that observed in male *Cgl* null mice and at a level indistinguishable from those observed in both sexes of a *Cbs* null mouse model of classical homocystinuria (HCU) that incurs severe liver damage (Maclean et al., 2010a). Furthermore, we report that compared to males, female *Cgl* null mice incur significantly increased expression of betaine homocysteine S-methyltransferase (BHMT) and methionine adenosyltransferase 1, alpha (MAT1A). Severely elevated tHcy in female *Cgl* null mice was found to be associated with a sex-specific 70% decrease in expression of methionine synthase (MTR) that was accompanied by significantly decreased plasma methionine. Similarly, decreased plasma cysteine levels in female *Cgl* null mice were associated with female-specific dysregulation of cysteine dioxygenase (CDO) expression. Comparative histological assessment of livers from both sexes of cystathionine beta-synthase (CBS) and female *Cgl* null mice indicated that cystathionine may have significant therapeutic potential against liver-injury. Collectively, our data sheds new light on the regulation of methionine/folate and cysteine metabolism in the presence of severely elevated Hcy and illustrates the importance of considering sex when investigating biochemical and physiological sequelae in genetically engineered mouse models of inborn errors of metabolism.

## RESULTS

### *Cgl* null female but not male mice exhibit severely elevated levels of plasma tHcy

In order to more fully characterize the previously reported sex-differences in tHcy in *Cgl* null mice, we measured plasma levels of tHcy and cystathionine, in male and female WT and *Cgl* null mice (*n*=10 for each group) as described in the Materials and Methods section. Both male and female *Cgl* mice exhibited massive accumulation of plasma cystathionine compared to WT controls but showed no significant difference as a consequence of sex (*P*=0.1581). Male *Cgl* null mice showed an approximate 10-fold increase in plasma tHcy compared to WT controls at a level comparable to both male and female mice treated with the CGL inhibitor compound PPG (Fig. 1B) and essentially identical to levels reported previously for male mice from this model (Yang et al., 2008). Female *Cgl* null mice exhibited an approximate 45-fold increase in plasma tHcy compared to WT controls (*P*<0.0001). This level is approximately 3.5-fold higher than male *Cgl* null mice (*P*<0.0001) and is essentially equivalent to those reported in the *Cbs* null and HO mouse models of HCU (Maclean et al., 2010a,b). Given the unusual nature of this latter finding, these determinations were repeated in three additional cohorts of female *Cgl* null mice (*n*=5 for each). Every mouse in all three of these experimental groups exhibited a similar dramatic elevation in plasma tHcy.

### *Cgl* null mice exhibit female-specific alteration in the expression of enzymes involved in the transsulfuration of methionine

In order to investigate why *Cgl* null mice incur female-specific severe elevation of plasma tHcy, we performed western blotting analysis of the expression levels of the enzymes involved in transsulfuration of methionine to cysteine in both male and female *Cgl* null mice and sex-matched WT control mice (*n*=6 for each group). Additionally, in the interests of completeness, we investigated protein levels of glycine N-methyltransferase (GNMT) which catalyzes the synthesis of N-methylglycine (MG, also known as sarcosine), from glycine using S-adenosylmethionine (AdoMet) as a methyl donor.

**Fig. 2.**
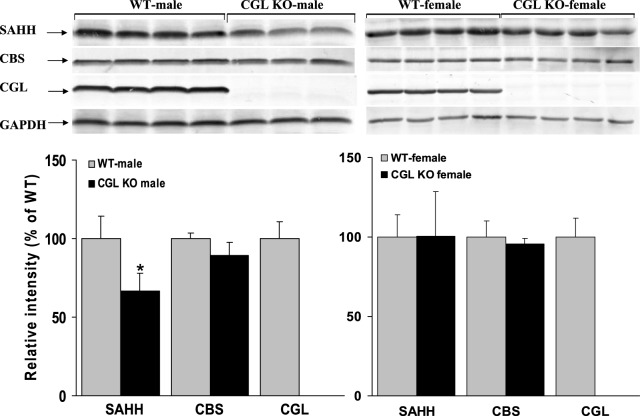
**Severely elevated Hcy does not induce CBS or SAHH expression in female *Cgl* null mice.** Western blotting analysis of hepatic SAHH, CBS and CGL protein expression levels in male and female WT and *Cgl* null mice. Blotting and immunostaining were performed as described in the Materials and Methods section, In this experiment and all subsequent blots, the relative intensities of protein bands were quantified using Quantity One version 4.6.5 software (Bio Rad). Signal intensity from target bands were calculated relative to GAPDH signal intensity. All blots shown are representative of a minimum of two independent experiments. Values shown represent the mean±s.d., *P<0.05.

As expected, we observed no trace of CGL protein in either male or female *Cgl* null mice (Fig. 2). This finding was subsequently confirmed by enzyme assay which found no detectable CGL activity confirming that our findings are not an artifact of erroneous genotyping. No significant increase in CBS protein was observed compared to WT controls in either female or male *Cgl* null mice (Fig. 2) indicating that this enzyme is not induced by elevated tHcy in either male or female *Cgl* null mice. Consistent with the western blotting analysis, enzyme assays found no significant difference in hepatic CBS activity between male and female WT and *Cgl* null mice (data not shown). Collectively, these results are consistent with the absence of any significant difference in plasma cystathionine between male and female *Cgl* null mice described above.

**Fig. 3.**
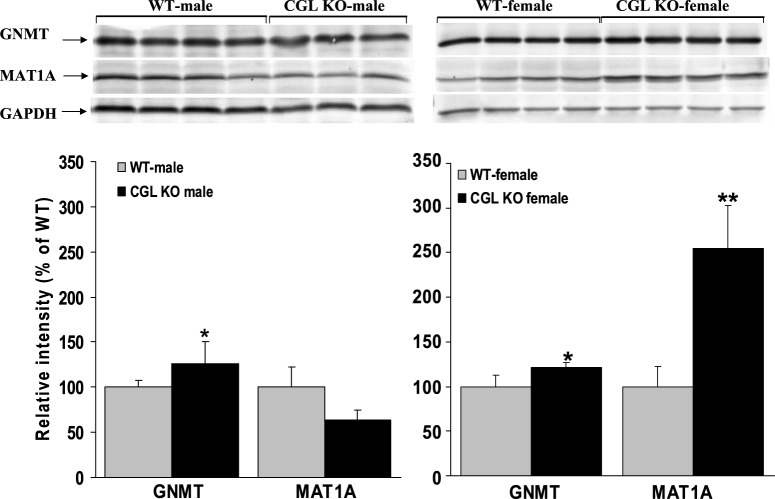
**MAT1A protein levels are significantly increased in female *Cgl* null mice with severely elevated plasma tHcy.** Western blotting analysis of hepatic GNMT and MAT1A protein expression levels in male and female WT and *Cgl* null mice. Values shown represent the mean±s.d., *P<0.05, **P<0.01.

Expression of S-adenosylhomocysteine hydrolase (SAHH) in male *Cgl* null mice was 40% lower compared to male WT controls (*P*=0.021) but was unaffected in female *Cgl* null mice (Fig. 2). Conversely, MAT1A was not significantly changed compared to controls in male *Cgl* null mice but was increased by approximately 2.5-fold in *Cgl* null female mice (*P*=0.0032) (Fig. 3). Interestingly, GNMT was significantly induced albeit at a relatively modest level, in both male and female *Cgl* null mice (*P*=0.0168 and 0.0108 respectively) (Fig. 3).

### *Cgl* null mice exhibit female-specific dysregulation of enzymes involved in the remethylation of methionine

In the mammalian liver, Hcy can be remethylated back to methionine by two distinct pathways. MTHFR catalyzes the irreversible reduction of 5,10-methylenetetrahydrofolate to 5-CH3-tetrahydrofolate (5-CH3-THF), the primary circulatory form of folate, which is subsequently used by MTR to remethylate Hcy to form methionine and tetrahydrofolate (THF) (Fig. 1A). An alternative pathway for hepatic Hcy remethylation involves BHMT which catalyzes the remethylation of Hcy to methionine using betaine as a methyl donor (Fig. 1A).

**Fig. 4.**
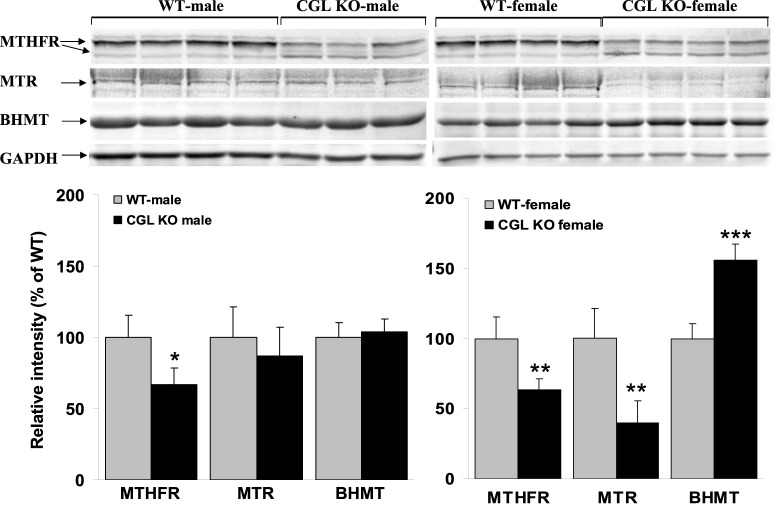
***Cgl* null mice exhibit female-specific alteration of enzymes involved in the remethylation of methionine.** Western blotting analysis of hepatic BHMT, MTHFR and MTR protein expression levels in male and female WT and *Cgl* null mice. Values shown represent the mean±s.d., *P<0.05, **P<0.01, ***P<0.001.

To further characterize Hcy metabolism in *Cgl* null mice, we examined the expression levels of BHMT, MTHFR and MTR in male and female WT and *Cgl* null mice (Fig. 4). In this analysis, we observed a significant decrease in MTHFR protein levels in male and female *Cgl* null mice relative to WT controls (*P*=0.048 and *P*=0.005 respectively). Previous work has shown that phosphorylation of the N-terminus of MTHFR results in increased catalytic activity and decreased sensitivity to the inhibitory effects of AdoMet (Yamada et al., 2005). The activated phosphorylated form of MTHFR can be resolved from the non-phosphorylated form by SDS-PAGE and we observed that both male and female *Cgl* null mice exhibit a similar decrease in the level of phosphorylated MTHFR as a percentage of total MTHFR protein (Fig. 4).

**Fig. 5.**
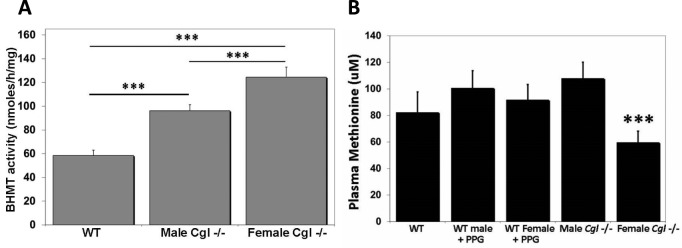
**BHMT activity is increased in *Cgl* null mice.** (A) Hepatic BHMT activity in WT control and male and female *Cgl* null mice was determined as described in the Materials and Methods. As there was no significant difference in BHMT activity between male and female WT mice values derived from both sexes were combined and represent an *n* of 12.Values shown for *Cgl* null mice represent the mean±s.d. derived from an *n* of 6 for each group. (B) *Cgl* null female but not male mice exhibit significantly reduced levels of plasma methionine. Plasma levels of methionine in wild type (WT) mice plus and minus treatment with the CGL specific inhibitor PPG and untreated male and female *Cgl* null mice. Values shown represent the mean±s.d. derived from 20 animals for WT and 10 animals for all other groups. ***P<0.001.

BHMT protein levels were unchanged in male *Cgl* null mice but were increased by approximately 30% in female *Cgl* null mice relative to WT controls (*P*=0.0003) (Fig. 4) suggesting a possible induction by elevated tHcy. This induction was subsequently confirmed by BHMT activity assays performed using liver extracts as described in the Materials and Methods section (Fig. 5A). This analysis observed a 40% and two-fold induction of BHMT activity in male and female *Cgl* null mice respectively (*P*=0.0006 and *P*=0.0039 respectively).

Compared to WT control mice, MTR protein levels were observed to be unchanged in male *Cgl* null mice but were dramatically reduced by approximately 70% in female *Cgl* null mice (*P=*0.004). Given the crucial role that MTR plays in regulating Hcy levels, the repression of MTR is the most likely cause of the female-specific severely elevated tHcy observed in *Cgl* null mice.

### *Cgl* null female but not male mice exhibit significantly reduced levels of plasma methionine

In HCU, severely elevated Hcy is invariably accompanied by significantly elevated methionine (Maclean et al., 2002a). Conversely, homocystinuria due to either MTHFR or MTR deficiency result in significantly lower levels of methionine compared to normal subjects. To assess the effect of decreased MTR levels in *Cgl* null mice, we determined the plasma levels of methionine in male and female WT and *Cgl* null mice. Male *Cgl* null mice showed essentially identical methionine levels compared to both male and female WT controls in both the presence and absence of PPG treatment. Consistent with the observed decrease in MTR expression described above, female *Cgl* null mice exhibited significantly lower plasma methionine compared to both WT control mice and *Cgl* male mice (*P*=0.0002 and *P*<0.0001 respectively) (Fig. 5B).

### *Cgl* null female but not male mice exhibit significantly reduced levels of plasma total cysteine

Previous work in humans and mice has shown that HCU is associated with significantly decreased plasma total cysteine (tCys) (Jiang et al., 2014; Maclean et al., 2002a, 2012b, 2010b). This decreased level of cysteine has typically been presumed to be due to the block in endogenous synthesis of this compound due to inactivating mutations in CBS. However, previous work in our laboratory has observed that lowering tHcy with betaine results in significantly increased plasma tCys (Maclean et al., 2012b, 2010b). As betaine treatment does nothing towards restoring transsulfuration, these finding argue that the diminished cysteine in untreated HCU may be a consequence of elevated tHcy. In this context, the male and female *Cgl* null mice which, are equally blocked in endogenous cysteine synthesis and ingesting identical levels of dietary cysteine, offer a unique opportunity to investigate the effect of severely elevated tHcy upon plasma tCys levels.

**Fig. 6.**
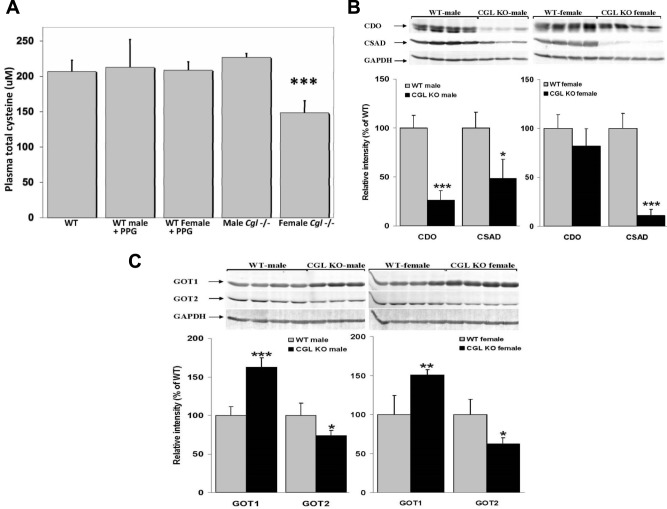
**Sex-specific alterations in cysteine metabolism in *Cgl* null mice.** (A) *Cgl* null female but not male mice exhibit significantly reduced levels of plasma cysteine. Plasma levels of cysteine in wild type (WT) mice plus and minus treatment with the CGL specific inhibitor PPG and untreated male and female *Cgl* null mice. *n*=10. (B,C) Male and female *Cgl* null mice exhibit sex-specific alterations in cysteine oxidation*.* Western blotting analysis and protein expression levels of hepatic CDO and CSAD (B) and GOT1 and GOT2 (C) in male and female WT and *Cgl* null mice. Values shown represent the mean±s.d., *P<0.05, **P<0.01, ***P<0.001.

We determined the plasma levels of cysteine in male and female WT mice plus and minus PPG treatment and male and female *Cgl* null mice. Male *Cgl* null mice showed essentially identical tCys levels compared to both male and female WT controls irrespective of PPG treatment (Fig. 6A). Interestingly, female *Cgl* null mice exhibited significantly lower plasma tCys compared to WT control mice plus or minus PPG (*P*=0.001) and *Cgl* male mice indicating that plasma levels of this compound are significantly lowered as a consequence of severely elevated tHcy rather than as a consequence of blocked endogenous synthesis.

### Male and female *Cgl* null mice exhibit sex-specific alterations in cysteine oxidation

In mammals, the main determinant in regulating plasma and tissue levels of cysteine is CDO, an iron (Fe^2+^)-dependent thiol dioxygenase that adds molecular oxygen to the sulfur of cysteine, converting the thiol to a sulfinic acid known as cysteinesulfinate (also known as 3-sulfinoalanine) which can be processed to either hypotaurine by CSAD or 3-sulfinylpyruvate by GOT1/2 (also known as aspartate aminotransferase) (Stipanuk and Ueki, 2011) This latter enzyme exists in both a cytosolic (GOT1) and mitochondrial (GOT2) isoforms (Stipanuk and Ueki, 2011) (Fig. 1A). In order to further characterize the possible sex-specific metabolic differences in *Cgl* null mice, we used western blotting analysis to investigate the expression levels of CDO, CSAD (Fig. 6B) GOT1 and 2 (Fig. 6C) in male and female WT and *Cgl* null mice.

In this analysis, we observed no significant difference in the levels of either CDO or CSAD between male and female WT mice. Male *Cgl* null mice repressed expression of CDO by approximately 80% (*P*<0.0001). Conversely, despite exhibiting significantly diminished plasma tCys, female *Cgl* null mice did not significantly lower CDO expression relative to either male or female WT controls. Both male and female *Cgl* null mice exhibited significantly decreased levels of CSAD relative to their respective WT controls (*P*=0.012 and *P*<0.0001 respectively) but the level of repression was significantly greater in female *Cgl* null mice compared to male mice (*P*=0.014). In the same animals, hepatic GOT1 expression was increased in both male and female *Cgl* null animals relative to WT controls (*P*<0.0001 and *P*<0.007 respectively). GOT2 showed the opposite pattern and was significantly repressed in both male and female *Cgl* null mice (*P*=0.046 and *P*=0.012 respectively).

### The hepatic phenotype of female *Cgl* null mice is consistent with a possible protective role for cystathionine against liver injury in *Cgl* null mice

**Fig. 7.**
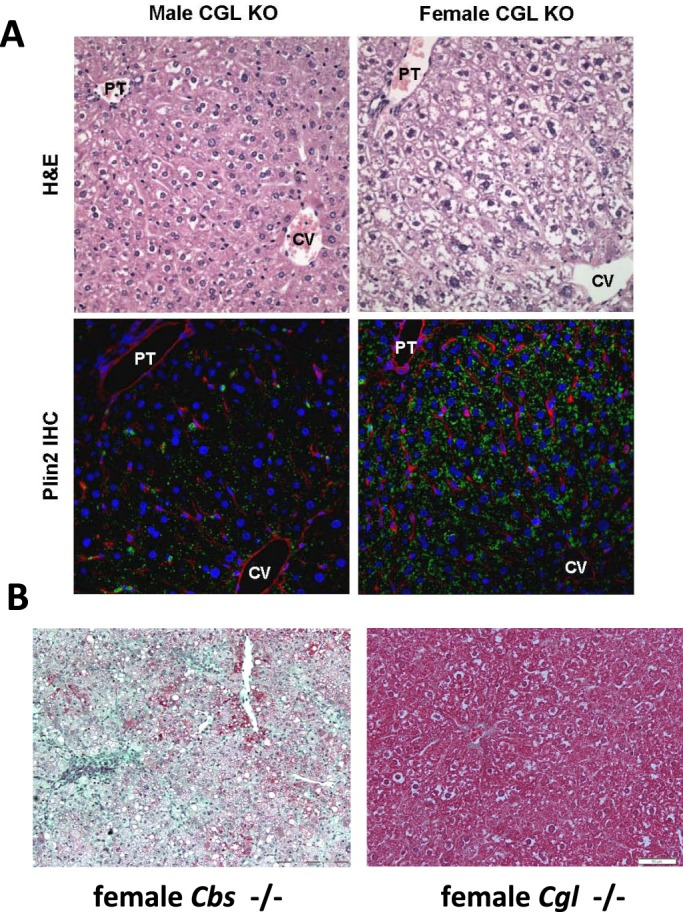
**The hepatic phenotype of female *Cgl* null mice is consistent with a possible protective role for cystathionine against liver injury in *Cgl* null mice.** (A) H&E (upper panels) and Plin2 immunohistochemical staining (lower panels) from representative liver sections from male and female *Cgl* null mice reveals mild levels of lipid accumulation and Plin2 expression (green signal) in female but not male *Cgl* null mice. Data shown is representative of 5 animals per group. PT, portal triad; CV, central vein. (B) Masson-trichrome staining of female *Cbs* and *Cgl* null mice with similar levels of plasma tHcy. No fibrosis was observed in any *Cgl* null (right panel) mouse sample. Photomicrographs shown are representative of staining from 20 views taken from 5 mutant mice and 5 wild type controls and were assessed by a pathologist without knowledge of genotype. Scale bars: 200 µm.

A previously described *Cbs* null mouse model of HCU incurs severe liver injury with approximately 90% of mice dying of liver failure in the first two weeks of life (Watanabe et al., 1995). Mice that survive the early neonatal period typically present with severe liver injury including panlobular microsteatosis, fibrosis, macrophage infiltration and severely elevated levels of plasma ALT (Maclean et al., 2010a). Previous work in our laboratory has indicated that elevated cystathionine acts to protect the liver of the HO mouse model of HCU and that this compound can exert significant protective effects against endoplasmic reticulum stress induced liver and kidney damage in WT mice (Maclean et al., 2012a, 2010b). The presence of similar levels of tHcy in the female *Cgl* null mice to those observed in *Cbs* null and HO HCU mice prompted a histological and immunohistochemical examination of male and female *Cgl* null mouse livers. In this analysis, male *Cgl* null mice exhibited no evidence of hepatic dysfunction with no detectable steatosis, fibrosis or inflammation (Fig. 7). ALT values for all male *Cgl* null mice investigated were within the normal range. H&E staining revealed that female *Cgl* null mice exhibit very low levels of hepatic steatosis. This finding was confirmed by staining for the cytoplasmic lipid droplet protein Plin2 (Fig. 7A). Masson trichrome staining of female *Cgl* null mice revealed no evidence of hepatic fibrosis (Fig. 7B) and plasma ALT levels were within the normal range. Collectively, our data indicates that female *Cgl* null mice incur minimal liver damage compared to *Cbs* null mice with similar levels of tHcy and are consistent with a possible hepatoprotective role for elevated cystathionine against liver injury that merits further investigation.

## DISCUSSION

Although relatively subtle changes in plasma tHcy as a consequence of sex hormone metabolism been reported previously (Giltay et al., 1998; Mantueffel-Cymborowska et al., 1992; Oktenli et al., 2003), to our knowledge, the *Cgl* null mouse model is the only reported case of profound sex-specific alterations in Hcy metabolism and serves to highlight the fact that sex is rarely considered in animal models of inborn errors of metabolism. This tendency is likely to be due to the need to conserve female mice for breeding particularly in mutant strains where fecundity is an issue. There is currently very little data available regarding metabolite levels in male and female patients with CGL deficiency but given that this condition is regarded as essentially benign (Kraus et al., 2009) it is unlikely that female cystathionuric patients exhibit the pathogenic levels of tHcy seen in female *Cgl* null mice. Currently, the reasons for the female-specific alterations of MTR, CDO and CSAD expression in female *Cgl* null mice are unclear. It is unlikely to be a generalized feature of impaired transsulfuration as although previous work in our laboratory has reported some differences in scale, we observed an essentially identical pattern of regulation of the cysteine oxidation enzymes and indistinguishable levels of plasma tHcy between male and female HCU mice (Jiang et al., 2014; Maclean et al., 2012b). Experiments designed to investigate the role of female and male sex hormones on the regulatory changes observed in *Cgl* null mice are currently ongoing in our laboratory.

The data presented in this report, indicate that female *Cgl* null mice are of questionable utility for studying the role of H_2_S in regulating vascular tone for two main reasons. Firstly, multiple investigators have previously published data indicating that mild elevations of tHcy in heterozygous *Cbs*^+/−^ mice result in impaired endothelial function in the vasculature by impairing the synthesis and bioavailability of nitric oxide (Upchurch et al., 1997). As male and female *Cgl* null animals exhibit tHcy levels some 8- and 23-fold higher than the *Cbs*^+/−^ animals respectively, there is a very strong possibility that this compound could cofound studies on vascular function. Secondly, the severely elevated tHcy observed in Female *Cgl* null mice appears to result in significantly decreased levels of cysteine which is the substrate for multiple desulfuration reactions involved in the synthesis of H_2_S. The sex-specific effects of severely increased tHcy and cystathionine in the presence of decreased cysteine levels on endothelial gasotransmitter production (e.g. NO and H_2_S) remains to be elucidated.

Female *Cgl* null mice may be a useful tool for examining homocystinuria due to MTR deficiency and the physiological consequences of vitamin B12 deficiency with regard to the methylfolate trap hypothesis. This latter hypothesis proposes that vitamin B12 deficiency impairs overall folate metabolism because the generation of 5-CH3-THF is irreversible and that impairment of MTR prevents conversion of this compound to THF resulting in significant accumulation of 5-CH3-THF and depletion of THF. In this context, it would be interesting to investigate if female *Cgl* null mice exhibit the hematological sequelae, cellular folate loss because of shorter 5-CH3-THF polyglutamate chains and global DNA hypomethylation that have previously been attributed as a consequence of the methylfolate trap.

Cobalamin C (CblC) disease is the most common inherited disorder of vitamin B_12_ metabolism, and has been ascribed to mutations in the methylmalonic aciduria and homocystinuria type C protein (*MMACHC*) gene that result in downstream deficiencies in methylcobalamin and adenosylcobalamin, which serve as enzymatic cofactors for MTR and methylmalonyl-CoA mutase (MCM) respectively. MCM is a mitochondrial enzyme that catalyzes isomerization of methylmalonyl-CoA to succinyl-CoA and its impairment results in methylmalonic acidemia. As a result of impaired MTR and MCM function, CblC disease results in severely elevated Hcy, methylmalonic acid and decreased methionine. Interestingly, CblC disease exhibits a number of unique clinical sequelae that are not present in HCU or methylmalonic acidemia despite having biochemical markers in common with both conditions (Weisfeld-Adams et al., 2013). MTR null mice survive through implantation but die shortly thereafter while MMAHC null embryos only survive to embryonic day 3.5 (Moreno-Garcia et al., 2014; Swanson et al., 2001). Consequently, the female *Cgl* null mice may offer a rare opportunity to study the pathological and metabolic effects of severely elevated Hcy and decreased methionine in isolation from severely elevated methylmalonic acid.

Regulation of MTHFR activity is crucial for maintaining cellular concentrations of methionine and AdoMet. Previous work has shown that the N-terminal extension of MTHFR contains a highly conserved serine-rich region that is phosphorylated and that dephosphorylation results in increased catalytic activity and decreased sensitivity to the inhibitory effects of AdoMet (Yamada et al., 2005). This group also presented data indicating that phosphorylation of MTHFR was influenced by intracellular AdoMet/AdoHcy levels which could be influenced by methionine depletion in female *Cgl* null mice. However, this possibility is unlikely as both male and female *Cgl* null mice exhibited reduced levels of total MTHFR protein and a similar shift towards the unphosphorylated form of the enzyme. Further work is required to see if these regulatory changes in MTHFR expression are unique to *Cgl* null mice or also occur in mice with elevated tHcy due to CBS deficiency.

Although in this study we have not examined oxidative stress parameters, the failure of severe elevations of the pro-oxidant Hcy and a concomitant decrease in the antioxidant cysteine to induce hepatic expression of CBS is consistent with previous reports that induction of this gene is regulated by the redox sensitive transcription factors Sp1 and Sp3 (Ge et al., 2001; Maclean et al., 2004) and that despite its crucial role in antioxidant synthesis, oxidative stress does not induce CBS expression (Maclean et al., 2002b).

Given the severely elevated levels of tHcy and concomitant decrease in methionine, it is clear that the relatively modest induction of BHMT protein and activity in female *Cgl* null is insufficient to compensate for the decrease in MTR protein levels in those mice. In this context it is interesting to note that that severely elevated Hcy in the HO mouse model of HCU results in an approximate three-fold reduction of BHMT protein levels and activity (Maclean et al., 2012b). A possible key metabolite in the apparent divergent regulatory response of BHMT expression to severely elevated Hcy in HCU mice and *Cgl* null female mice could be methionine. However, this compound has to date been regarded as an inducer of BHMT expression but is significantly reduced in the female *Cgl* null animals and strongly elevated in both the HO and *Cbs* null mouse models of HCU (Maclean et al., 2010a,b).

The female-specific decrease in methionine in *Cgl* null mice appears to be due to a 70% decrease in expression of MTR. Given that HO HCU mice exhibit decreased BHMT expression and strongly elevated methionine levels it is highly unlikely that this repression of MTR occurs in HCU. Consequently, it is conceivable that either accumulation of 5-CH3-THF or decreased THF levels as a consequence of the repression of MTR and MTHFR could be exerting previously unsuspected regulatory effects upon BHMT expression in female *Cgl* null mice. As the product of the BHMT reaction with betaine and Hcy produces DMG which is subsequently converted to MG and then glycine in reactions that both involve the formation of 5,10-CH2-THF from THF it is clear that further work is required to fully elucidate the regulation of BHMT expression in the presence of severely elevated Hcy due to decreased MTR activity. Such an investigation may offer some clues as to why betaine therapy is relatively ineffective in patients with defective synthesis of methylcobalamin (Allen et al., 1993).

CBS and CGL are both essential enzymes for the endogenous synthesis of cysteine from Hcy in mammals. CDO is well documented as a crucial regulator of plasma and tissue levels of cysteine and in previous work with the HO mouse model of HCU, we have shown that decreased cysteine levels are accompanied by decreased CDO protein levels in an apparent attempt to conserve cysteine (Jiang et al., 2014). This observation is consistent with previous reports from a large body of work investigating the regulation of CDO by the Stipanuk laboratory (Stipanuk and Ueki, 2011). Consequently, the normal levels of cysteine in male *Cgl* null mice may result at least in part, from the repression of hepatic CDO expression acting to conserve tissue levels of this compound. Similarly, previous analysis of CSAD in HO HCU mice in our laboratory found this enzyme was strongly induced in HO mice in proportion to the degree of Hcy elevation and that this induction could be reversed by either taurine or cysteine supplementation (Jiang et al., 2014). In contrast to these findings, we observed CSAD was repressed in both male and female *Cgl* null mice. Consequently, it will be interesting to see what effects cysteine and taurine supplementation have upon CSAD expression levels in *Cgl* null mice.

Previous observations of diminished cysteine levels in CBS deficiency have typically been attributed to a block in this pathway. However, this mechanism is unlikely to be responsible for decreased tCys levels as lowering tHcy in HCU with either betaine treatment or methionine restriction results in restoration of plasma tCys levels without restoring endogenous biosynthesis (Gupta et al., 2014; Maclean et al., 2012b, 2010b). In this context, the observation of significantly decreased plasma cysteine levels in female but not male *Cgl* null mice is interesting as both sexes are equally blocked in endogenous cysteine biosynthesis. These findings are consistent with the hypothesis that it is the severely elevated tHcy that is responsible for the observed decrease in cysteine rather than the impairment of transsulfuration. One possible mechanism where severely elevated tHcy could result in decreased plasma cysteine is by the formation of mixed disulfides with cysteine and subsequent excretion in the urine. This possibility is currently the subject of investigation in our laboratory.

In conclusion, much of what we know about regulation of the methionine/folate cycle and cysteine oxidation has been determined in wild type animals. Previous work in our laboratory on the regulation of BHMT, CDO and CSAD (Jiang et al., 2014; Maclean et al., 2012b) in HCU and our findings described here, indicate that it is not axiomatic that those regulatory principles will be conserved in diseases where genes involved in that metabolism are inactivated. Collectively, our findings indicate that it would be wise to compare the metabolic data between males and females during the initial characterization of any mouse model of an inborn error of metabolism and that the rational design of treatments for that disease would benefit from a comprehensive assessment of all the relevant enzymes that might be perturbed.

## MATERIALS AND METHODS

### Chemicals and reagents

Unless otherwise stated, all chemicals were obtained from Sigma. BHMT-specific antisera (#ARP41474_T100) was obtained from Aviva systems Biology Corporation. CDO (#ab53436) and cysteinesulfinate decarboxylase (CSAD; #ab91016) antisera were obtained from Abcam, glutamic-oxaloacetic transaminase 1(GOT1; #AAS05482C) and glutamic-oxaloacetic transaminase 2 (GOT2; #AAS17435C) antibodies were obtained from Antibody Verify. Antibodies for CBS (#H00000875-M06), CGL (#H00001491-M02) and SAHH, (#H00000191-B01) were obtained from Abnova. Antibodies for MTR (#NB100-791) and MAT1A (NBP1-55120) were obtained from Novus biologicals. Glycine N-methyltransferase (GNMT; #sc-68871) specific antibody was obtained from Santa Cruz Biotechnology. MTHFR specific antibody was a generous gift from Professor Rima Rosen (McGill University, Montreal Canada). Perilipin 2 (Plin2; #20R-AP002) specific antibody was obtained from Fitzgerald Industries International. Glyceraldehyde 3-phosphate dehydrogenase (GAPDH; #A300-641A) antibody was obtained from Bethyl laboratories.

### Animal experiments diet and treatments

C57/Bl6 virgin female mice (10 to 12 weeks) were purchased from Charles River Laboratories and housed in the University of Colorado Anschutz Medical campus vivarium. *Cgl* null mice were a kind gift from Professor Solomon H. Snyder (Dept. of Neuroscience, Johns Hopkins School of Medicine, Baltimore, MD). Mice were genotyped as described previously (Yang et al., 2008) and were housed in individual cages on a 12 h light/dark cycle at a mean temperature of 22°C. All mice were maintained on standard chow (LabDietNIH5K67, PMI nutrition international, Brentwood, MO). There was no significant difference in body weight or food intake observed between male and female *Cgl* null mice. *Cgl* null mice were backbred through 6 generations of C56/Bl6 mice, and animals were bred through heterozygous crosses on a C57/Bl6 background (Charles River) at least once every 3–6 months. We have not observed any changes in the metabolic phenotype of these animals over 2 years of breeding in our facility.

Experimental hypercystathionemia in wild type (WT) C57/Bl6 mice was induced by intra-peritoneal injection of the CGL inhibitor propargylglycine (PPG, 50 mg day^−1^ kg^−1^) as described previously (Barber et al., 1999). All experiments were approved by the University of Colorado Health Sciences Center institutional animal care and use committee and were performed according to the NIH standards for animal care and use.

### Thiols and methionine cycle metabolites and enzyme activity assays

Determination of plasma levels of methionine cycle metabolites was performed as described previously (Stabler et al., 1993). CBS, CGL and BHMT activity assays were performed as described previously (Galligan et al., 2012; Mulligan et al., 1998).

### Histological examination of tissues and assessment of hepatopathy

Tissues were immersion-fixed overnight in 4% paraformaldehyde in PBS (pH 7.3). Paraffin embedded sections (5 μm) were stained for examination with hematoxylin and eosin (H&E). Liver sections were stained for the presence of Plin2 using commercial antibodies against Plin2 (1:300, diluted in 10% normal goat serum in PBS) plus fluorescently labeled secondary antibody or horse radish peroxidase labeled secondary antibody and the chromogenic substrate diamino-benzidine. Nuclei were stained with DAPI. All slides were assessed independently by two observers blinded to genotype. Liver injury was further assessed by determining plasma levels of alanine aminotransferase (ALT) activity using an enzyme-coupled assay with lactic dehydrogenase as described previously (Bergmeyer and Horder, 1980).

### Preparation of liver homogenates for Western blotting

Liver samples were homogenized in buffer containing 100 mM KPi (pH 7.4), 1 mM EDTA and 1:50 (v/v) protease inhibitor cocktail from Sigma. The ratio of liver tissue to lysis buffer was 1 g of liver tissue to 5 ml of lysis buffer. The homogenate was subsequently centrifuged at 4°C at 20,000 ***g*** for 20 min and the supernatant thus formed, was used as a crude extract. The protein concentration of crude extracts was determined by the Bradford method using bovine serum albumin as a standard (Bradford, 1976).

### SDS-PAGE and western blotting analysis

Immunoblot analysis of total liver lysates was performed as described previously (Jiang et al., 2012). Anti-sera were used at a dilution of either 1:500 (GNMT), 1:1000 (BHMT, MTR and MAT1A), 1:2000 (CBS, CGL, CDO, CSAD, SAHH, GOT1 and GOT2) or 1:5000 (MTHFR and GAPDH). Signals were detected using a Typhoon 9400 system (Amersham Pharmacia) after incubation with appropriate Fluorescein- or Texas red-conjugated secondary antibodies (Vector Laboratories), or Alexa Fluor 647-conjugated secondary antibody (Invitrogen) of 1:2500 (v/v). The relative intensities of protein bands were quantified by software Quantity One version 4.6.5 software (Bio Rad). Signal intensities from target protein bands were calculated relative to GAPDH signal intensities in liver homogenates.

### Statistical analysis

All data are presented as means±s.d. and were compared using the unpaired Student's *t*-test. A *P* value of less than 0.05 was considered statistically significant. In the graphed data *, **, and *** denote *P* values of <0.05, 0.01, and 0.001 respectively.
